# Extract the Relational Information of Static Features and Motion Features for Human Activities Recognition in Videos

**DOI:** 10.1155/2016/1760172

**Published:** 2016-08-29

**Authors:** Li Yao

**Affiliations:** ^1^Key Laboratory of Computer Network and Information Integration (Southeast University), Ministry of Education, Nanjing, Jiangsu Province, China; ^2^State Key Laboratory for Novel Software Technology, Nanjing University, Nanjing, Jiangsu Province, China

## Abstract

Both static features and motion features have shown promising performance in human activities recognition task. However, the information included in these features is insufficient for complex human activities. In this paper, we propose extracting relational information of static features and motion features for human activities recognition. The videos are represented by a classical Bag-of-Word (BoW) model which is useful in many works. To get a compact and discriminative codebook with small dimension, we employ the divisive algorithm based on KL-divergence to reconstruct the codebook. After that, to further capture strong relational information, we construct a bipartite graph to model the relationship between words of different feature set. Then we use a *k*-way partition to create a new codebook in which similar words are getting together. With this new codebook, videos can be represented by a new BoW vector with strong relational information. Moreover, we propose a method to compute new clusters from the divisive algorithm's projective function. We test our work on the several datasets and obtain very promising results.

## 1. Introduction

Recognizing human activities in video automatically is a promising technology in computer vision. There are a lot of application scenarios for it, such as content-based video retrieval, intelligent video surveillance, human-computer interaction, and e-health. Although lots of researchers have paid attention to this problem, it remains challenging to recognize human activities in the videos because of the great variance caused by illumination change, camera motion, and background cluster and so on.

To recognize the human activities pattern from massive videos, researchers extract discriminate features for further process. Below, we briefly review some works based on static local features and motion features.

Regarding local static features, Lowe [1] proposed a descriptor which is invariant to translations, rotations, and scaling transformation. This descriptor detects interest points from a grey-level image at which statistics of local gradient directions of image intensities were accumulated to give a summarizing description of the local image structures in a local neighborhood around each interest point. In this paper, we use a dense version of SIFT descriptor which has been proven to be useful for tasks such as object categorization, texture classification, image alignment, and biometrics [2]. On the other hand, to make use of color information in image, color-based sift descriptor has been proposed [3].

As for local motion features, to capture temporal information in videos, Chen and Hauptmann [4] proposed a Mo-SIFT descriptor that detects interest points and encodes their local appearance and explicitly models local motion. Wang et al. [5] proposed an approach to describe trajectories densely. Laptev et al. [6] proposed a STIP descriptor that computed each interest point's descriptors of the associated space-time patch.

However, local static features and local motion features only contain partial information of human activities in video. Moreover, we believe that local motion and static features are complementary for action recognition in unrestricted videos [7]. Researchers have paid attention to fusion multimodality for getting complementary information [8]. As a similar work to this paper, Liu et al. [7] first extract local static feature and local motion feature from videos. After that, they use static information to prune motion features and use Page-Rank to prune local static features. Moreover, they employ the divisive algorithm based on KL-divergence for code word clustering. And features are mixed up during the classification phrase.

A low dimension BoW may lose important relation information, while a high dimension may lead to the curse of dimensionality. So, reconstructing original codebook to a smaller dimension with less lost information is necessary. To solve this problem, Fulkerson et al. [9] used Informational Bottleneck to obtain meaningful feature clusters. And Pereira [10, 11] used distributional cluster words/features. Each word cluster can then be treated as a single feature and thus dimensionality can be drastically reduced.

In our work, we use a bimodel to capture hybrid features before the classification phrase. We do it with two reasons: (1) capturing relational information in a direct way and (2) reducing the BoW dimensionality apparently.

As our first contribution, we employed the divisive algorithm based on KL-divergence. This algorithm uses divergence to generate an information loss criterion and is implemented iteratively like *k*-means. This makes it possible to capture compact and discriminative codebook with smaller dimension effectively and efficiently.

As our second contribution, we initiate employment of bimodel to get hybrid feature representation. We construct a bipartite graph to model the relationship between codebooks of different feature set. Then, we use a *k*-way partition to get a new codebook. With this new codebook, videos can be represented by a new BoW vector with strong relational information. A similar work [12] employs bimodel to get joint audiovisual codebook. The bimodel needs the clusters. We propose a method to compute new clusters from the divisive algorithm's output, while Ye et al. [12] generate the new clusters directly.

## 2. Methods

**Figure 1:**
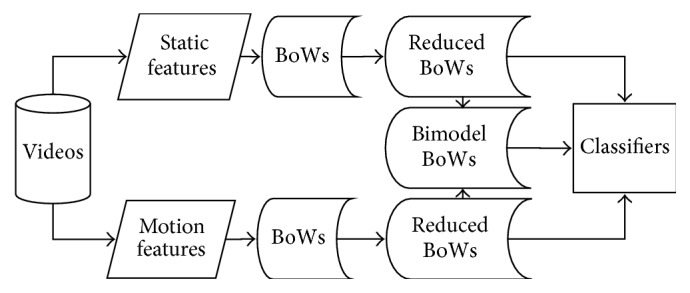
Flow chart of proposed method.

Figure 1 is the flowchart of the proposed system. In this paper, we propose using several technologies for better exploiting relational information of static feature and motion feature for human activities task.

### 2.1. Features Extraction and BoW Model

#### 2.1.1. Local Static Features

We detect local interest points by a Harris-Laplace detector densely and use a SIFT descriptor to encode these points. SIFT is invariant to rotation, scale, and light change. Moreover, the dense SIFT has been proven to be useful for tasks such as object categorization, texture classification, image alignment, and biometrics [2].

#### 2.1.2. Local Motion Features

In our work, we employ the Dense Trajectory descriptor [5] as original motion feature. Dense Trajectory firstly samples points in different spatial scale densely. After that, tracking is performed in the corresponding spatial scale. Finally, descriptors are computed along the trajectory. In this paper, we simply use the default parameters for feature extraction.

#### 2.1.3. BoW Model

BoW model has been widely used in many works and has been shown to be efficient in many tasks. This model clusters all features to several clusters and uses these clusters to discrete features from a video. Although BoW is effective and efficient, it may lose information with a low dimension or lead to curse of dimensionality with a high dimension. So, we will detail our codebook reconstruction technology in the next section for this problem.

### 2.2. Codebook Reconstruction

Although BoW model is efficient in many computer visual tasks, it has two obvious drawbacks. First, it is inevitable to lose information with low dimension; second, it may lead to the curse of dimensionality with high dimension. So, in this paper, we use a two-phrase procedure to get the BoW representation. Firstly, we use *k*-means to get a large codebook. Then, a divisive algorithm based on KL-divergence is employed to reconstruct the initial codebook to a small and discriminative codebook. To make use of bimodel, we propose a method to compute new clusters from projective function of original words.

#### 2.2.1. Compute Original Words' Projective Function

Suppose *p*_1_(*x*) and *p*_2_(*x*) are probability distributions taken from random variable *X*. The Kullback-Leibler (KL) divergence between *p*_1_(*x*) and *p*_2_(*x*) is defined as

(1) $$\begin{array}{c} KL\left(\;p_{1},p_{2}\;\right)=\underset{x\in X}{\sum }p_{1}\left(\;x\;\right)\log\frac{p_{1}\left(x\right)}{p_{2}\left(x\right)}. \end{array}$$

On the other hand, the Jensen-Shannon (JS) divergence is defined as

(2) $$\begin{array}{c} {JS}_{\pi }\left(\;p_{1},p_{2}\;\right)=\underset{i=1,2}{\sum }\pi_{i}KL\left(\;p_{i},\underset{j=1,2}{\sum }\pi_{j}p_{j}\;\right), \end{array}$$

where *π*_1_ + *π*_2_ = 1.

Let *C* = (*C*_1_,…, *C*_*m*_) represent activity classes, and *X* = (*x*_1_,…, *x*_*n*_) represent the original codebook. Then the information about *C* captured by *X* can be measured by mutual information *I*(*C*; *X*). Suppose *Y* = (*y*_1_,…, *y*_*k*_) is the new codebook we get; then we can measure the quality of the new codebook by the loss of MI, which is defined as

(3) QYIC;X−IC;Y=∑i=1kπyiJSpC ∣ xt:xt∈yi,

where *π*(*y*_*i*_) = ∑_*x*_*t*_ ∈ *y*_*i*__ *π*_*t*_, *π*_*t*_ = *p*(*x*_*t*_). And, after some derivation, we can rewrite *Q* as follows:

(4) $$\begin{array}{c} Q\left(\;Y\;\right)=\overset{k}{\underset{i=1}{\sum }}\pi \left(\;y_{i}\;\right)\underset{x_{t}\in y_{i}}{\sum }\pi_{t}*KL\left(\;p\left(\;C\;|\;x_{t}\;\right),p\left(\;C\;|\;y_{i}\;\right)\;\right). \end{array}$$

In this paper, we can use an iterative procedure like *k*-means algorithm to obtain the optimal new vocabulary using five major steps as follows:

(1) Perform initialization: for every original word *x*_*t*_, assign it to *y*_*j*_ (1 ≤ *j* ≤ |*C*|) with *j* = max⁡(*p*(*C*_*i*_∣*x*_*t*_)). After that, we get |*C*| initial word clusters. And then, each cluster is split to several groups which result in *k* initial clusters, say *Y* = (*y*_1_,…, *y*_*k*_).
(2) For each *y*_*j*_, compute
  (5) πyj=∑xt∈yjπt,PC ∣ yj=∑xt∈yjπtπyjpC ∣ xt.
(3) For each original word *x*_*t*_, assign it to another cluster *y*_*j*_, where *y*_*j*_ is
  (6) $$\begin{array}{c} j^{*}\left(\;x_{t}\;\right)=\underset{i}{\mathrm{argmin}}\;KL\left(\;p\left(\;C\;|\;x_{t}\;\right),p\left(\;C\;|\;y_{i}\;\right)\;\right). \end{array}$$
(4) Compute the measurement *Q*:
  (7) $$\begin{array}{c} Q\left(\;Y\;\right)=\overset{k}{\underset{i=1}{\sum }}\pi \left(\;y_{i}\;\right)\underset{x_{t}\in y_{i}}{\sum }\pi_{t}*KL\left(\;p\left(\;C\;|\;x_{t}\;\right),p\left(\;C\;|\;y_{i}\;\right)\;\right). \end{array}$$
(5) If *Q* < 10^−3^, exit the iteration; otherwise repeat steps (2) to (4).

We now discuss the computational complexity of our algorithm. Step (3) of each iteration requires KL-divergence to be computed for every pair, *p*(*C*_*j*_, *x*_*t*_) and *p*(*C*_*j*_, *y*_*i*_). This is the most computationally demanding task and costs a total of *O*(*mkl*) operations. Moreover, it can be proven that the objective function decreases at every iteration. So, the total time complexity is *O*(*mklt*), where *t* is the number of iterations.

#### 2.2.2. Compute New Clusters

Given the projection from original words to new words, we need to compute the clusters of the new words. To be specific, let *Y* = (*y*_1_,…, *y*_*k*_) be the new clusters; for each *y*_*i*_ in *Y*, we have

(8) $$\begin{array}{c} y_{i}=\frac{\sum_{x_{t}\in y_{i}}\left(x_{t}\times \sum_{j=1}^{n}d\left(j,t\right)\right)}{\sum_{x_{t}\in y_{i}}\sum_{j=1}^{n}d\left(j,t\right)}, \end{array}$$

where *n* is the number of training videos. And *d*(*j*, *t*) represent the entry *t* of the *j*th video.

### 2.3. Bimodel Based Relational Information Extraction

Given two codebooks extract from local static feature and local motion feature, we need to generate a new codebook which has as more relational information as possible. In this paper, we propose using a bimodel for this problem. Bimodel has been applied to IR [13] and Cross-View Action Recognition [14] successfully. To further capture strong relational information, we construct a bipartite graph to model the relationship between codebooks of different feature set. Finally, we use a *k*-way partition to get a new codebook. With this new codebook, videos can be represented by a new BoW vector with strong relational information.

#### 2.3.1. Construct a Bipartite Graph

In this section, we detail how to model the relationship between two words from two codebooks. Suppose we have *n* training videos named *D*_tr_^sta^ = {(*h*_*i*_^sta^, *l*_*i*_)}_*i*=1_^*n*^ and *D*_tr_^mot^ = {(*h*_*i*_^mot^, *l*_*i*_)}_*i*=1_^*n*^ captured from static feature and motion feature individually. Let *W* ^sta^ = {*w*_*i*_^sta^}_*i*=1_^*m*_sta_^ and *W* ^mot^ = {*w*_*i*_^mot^}_*i*=1_^*m*_mot_^ be the codebooks of static feature set and motion feature set individually. We can construct a graph *G* = (*V*, *E*), where *V* and *E* represent the vertices and edges, respectively. To be specific, as *G* is a bipartite graph, *V* = *V* ^sta^ ∪ *V* ^mot^, where each vertex in *V* ^sta^ corresponds to a static word in *W* ^sta^ and each vertex in *V* ^mot^ corresponds to a motion word in *W* ^mot^. Moreover, each edge in *E* only connects the vertices between *V* ^sta^ and *V* ^mot^. The weight matrix of *E* can be defined as $E=\left(\begin{array}{cc} 0 & S\\ S^{T} & 0 \end{array}\right)$, where *S* is a |*V* ^sta^| × |*V* ^mot^| matrix representing the similarity between any pair of words from two codebooks. In this paper, we use a measurement like TF-IDF to measure the similarity. To be specific, each element *s*_*kl*_ of *S* is defined as follows:

(9) $$\begin{array}{c} s_{kl}=\frac{\sum_{i=1}^{n}h_{i}^{sta}\left(k\right)h_{i}^{mot}\left(l\right)}{\sum_{i=1}^{n}h_{i}^{sta}\left(k\right)\sum_{i=1}^{n}h_{i}^{mot}\left(l\right)}, \end{array}$$

where *h*_*i*_^sta^(*k*) denotes the entry of *h*_*i*_^sta^ corresponding to static words *w*_*k*_^sta^ and *h*_*i*_^mot^(*l*) denotes the entry of *h*_*i*_^mot^ corresponding to motion words *w*_*l*_^mot^.

#### 2.3.2. Discover Bimodel Words

After obtaining the bipartite graph between static feature codebook and motion feature codebook, we present the detail of bimodel words discovery.

(*1) Graph Bipartitioning*. Given a bipartite graph *G* = (*V*, *E*), bipartitioning is to partition *V* into two subsets, where vertices in the same subset have strong relation and vertices in the different subset have weak relation. Formally, graph bipartitioning aims at minimizing the following objective function:

(10) $$\begin{array}{c} cut\left(\;V_{1},V_{2}\;\right)=\underset{i\in V_{1},j\in V_{2}}{\sum }s_{ij}. \end{array}$$

(*2) Efficient k-Way Solution*. Actually, finding bipartitioning of bigraph can be understood as classifying each point into two classes, for example, +1 and −1. Suppose *q*_*i*_ is the projection value of vertices *i*; good bipartitioning minimized (1/4)∑_(*i*,*j*)∈*E*_ *e*_*ij*_ × (*q*_*i*_ − *q*_*j*_)^2^. However, this may lead to a wrong solution that assigns all vertices to +1 or −1. So, in this paper, we are actually looking for a balanced partition whose objective function looks like the following:

(11) $$\begin{array}{c} Balance\;\;Cut\left(\;V_{1},V_{2}\;\right)=\frac{cut\left(V_{1},V_{2}\right)}{\sum_{i\in V_{1}}\sum_{j}e_{ij}}+\frac{cut\left(V_{1},V_{2}\right)}{\sum_{i\in V_{2}}\sum_{j}e_{ij}}. \end{array}$$

This problem can be solved by spectral clustering, which first constructs a Laplace matrix *L* as follows:

(12) $$\begin{array}{c} L\left(\;i,j\;\right)=\left\{\begin{array}{cc} -e_{ij} & e_{ij}\in E\\ \underset{k}{\sum }e_{ik} & i=j\\ 0 & else. \end{array}\right. \end{array}$$

After that, bipartitioning of *G* can be provided by the second smallest eigenvector of the generalized eigenvalue problem *Lz* = *λDz*, where *D*(*i*, *i*) = ∑_*j*_ *e*_*ij*_.

However, as an efficient solution proposed in [13], we can get optimal bipartitioning without computational complex. Suppose we have a matrix *L*, where *D*_1_^sta^(*i*, *i*) = ∑_*j*_ *e*_*ij*_ and *D*_2_^mot^(*i*, *i*) = ∑_*j*_ *e*_*ji*_, as follows:

(13) Li,jD1sta−S−STD2mot=D1sta00D2mot+0−S−ST0.

Let *𝕊* = *D*_1_^sta^ ^−1/2^ *SD*_2_^mot^ ^−1/2^; it can be proven that the second eigenvector of *L* can be expressed in terms of left and right singular vectors (say *u*_2_ and *v*_2_) of *𝕊* as follows:

(14) $$\begin{array}{c} z_{2}=\left(\begin{array}{c} {D_{1}^{sta}}^{-1/2}u_{2}\\ {D_{2}^{mot}}^{-1/2}v_{2} \end{array}\right). \end{array}$$

In a general scene, suppose we need to capture *k* new words containing relational information; the optimal *k*-way partitioning solution is provided by the *l* = ⌈log⁡*k*⌉ singular vectors *U* = (*u*_2_,…, *u*_*l*+1_) and *V* = (*v*_2_,…, *v*_*l*+1_).

To be specific, let *Z* = (*D*_1_^sta^ ^−1/2^ *U*, *D*_2_^mot^ ^−1/2^ *V*)^*T*^; we look for *k* clusters of row space in *Z* such that the sum of squares ∑_*i*=1_^*k*^∑_*j*_distance(*i*, *j*) is minimized.

Thus, our bimodel based clustering algorithm can be summarized as five basic steps as follows:

 
*Input: n* training videos.
 (1) Construct bipartite graph, where each element of *S* is computed as formula (9).
 (2) Compute matrices *D*_1_^sta^ and *D*_2_^mot^ and *𝕊* = *D*_1_^sta^ ^−1/2^ *SD*_2_^mot^ ^−1/2^.
 (3) Apply SVD on *𝕊*; compute *U* and *V*.
 (4) Compute matrix *Z*, whose size is (|*V* ^sta^| + |*V* ^dyn^|) × l.
 (5) Run *k*-means on row vectors of matrix *Z* to get *k* clusters.
 
*Output: k* clusters.

With *k* new clusters, each video can be represented as a new BoW vector which contains relational information.

## 3. Experiment and Analysis

### 3.1. Experiment on Olympic Dataset

**Figure 2:**
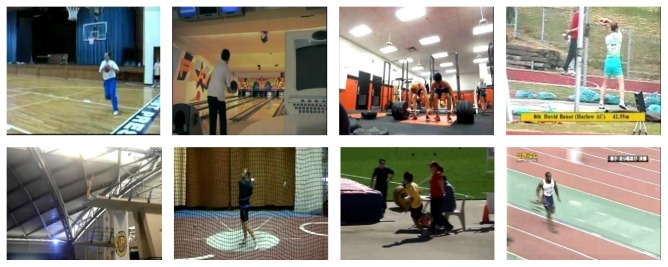
Screenshots of Olympic dataset.

The Olympic dataset (Figure 2) contains videos of athletes practicing different sports [15]. As all the videos are crawled from YouTube, it means that there are little artificial constraints which make the human activities recognition hard. There are 16 sports including high jump, long jump, and basketball. In our experiment, we use the default solution to split the training videos and testing videos. Figure 2 shows some screenshots from this dataset.

We use the package provided by [3] to extract dense SIFT features. For each video, we extract SIFT from the densely sampled grid with default parameters. In our experiment, we extract nearly 800000 SIFT features. We use the tool provided by [5] to extract Dense Trajectory features with default parameters. Finally, we sample every 100 frames and get about 60000 features for each event. After that, every video is represented by BoW vectors. And we use a grid search and 5-fold cross validation to get optimal parameters for SVM [16] classifiers.

To demonstrate the effectiveness of our method, we implement four experiments for comparison. In the first experiment, we simply used dense SIFT to recognize the testing videos. The second experiment only used Dense Trajectory for recognition. The third experiment extracted the relational information with bimodel. Finally, to demonstrate the influence of our codebook reconstruction method, we combine bimodel and the divisive algorithm we detailed before, which is called all-in-one algorithm.

**Figure 3:**
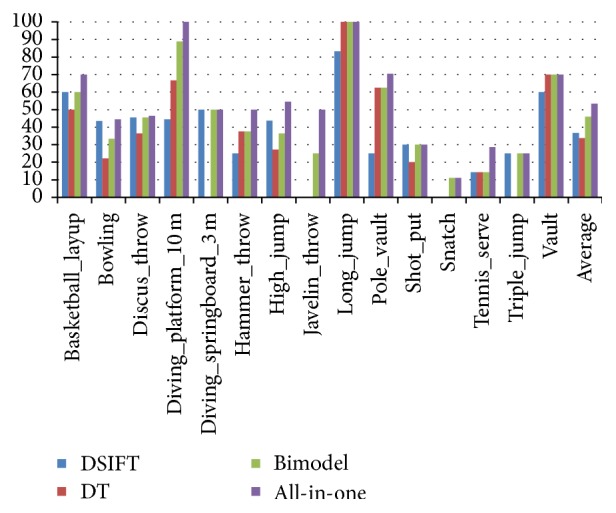
Experiment results of our experiment on 16 events of the Olympic dataset.

As Figure 3 shows, the average accuracy of bimodel is obviously higher than the dense SIFT [3] and Dense Trajectory [5]. And, in most of the cases, the bimodel based accuracy is higher than or the same as the other two features' accuracy. This is in accord with our intuition that relational information contains message from both features which results in better result. Moreover, as the number of testing videos in “javelin_throw” and “snatch” is very small, the single feature based classifiers perform badly. But the bimodel based classifiers can still deal with them. This is due to the fact that bimodel relational information contains more information that single feature does not include. Our experiments results show that the all-in-one algorithm is performing better than other three experiments in almost all cases.

### 3.2. Experiment on KTH Dataset

**Figure 4:**
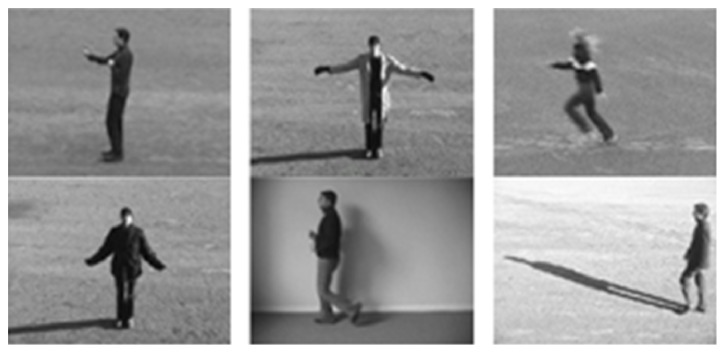
Screenshots of KTH dataset.

The KTH dataset [17] consists of six human action classes. Each action class is performed by 25 people. And every person repeats one action 4 times under different scenarios.  Figure 4 shows some screenshots from this dataset.

**Figure 5:**
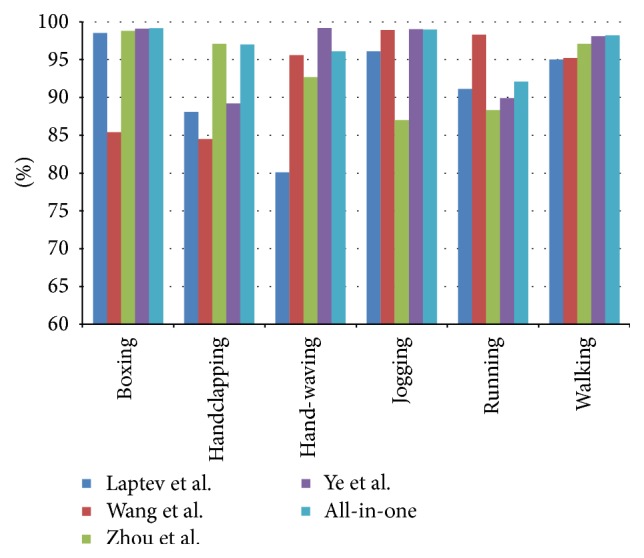
Compare proposed method with other BoW based methods on KTH dataset.

As Figure 5 shows, we compare our proposed all-in-one method with other state-of-the-art methods. Among them, Laptev et al. [6] used STIP descriptor. Wang et al. [5] used Dense Trajectory descriptor in multiple scales. And Ye et al. [12] proposed a joint audiovisual bimodal using SIFT and STIP features. Zhou et al. [18] proposed a novel structured codebook construction method to encode rich spatial and temporal contextual information for human action recognition.

It is shown that the proposed all-in-one method is better than other methods for the “boxing,” “hand-waving”, “jogging,” and “walking” actions. Meanwhile, we observe that proposed methods perform relatively worse in “handclapping” class and “running” class. Because the “running” action looks similar to the “jogging” action except the speed and the “handclapping” action looks similar to the “hand-waving” action, we need more specific information to distinguish them.

### 3.3. Experiment on TRECVID MED Dataset

TRECVID MED is a challenging task for the detection of complicated high-level events. We test our proposed method on the prespecified evaluation events in TRECVID MED 2016 development dataset [19], which includes 20 events. This dataset consists of 200,000 videos.

**Figure 6:**
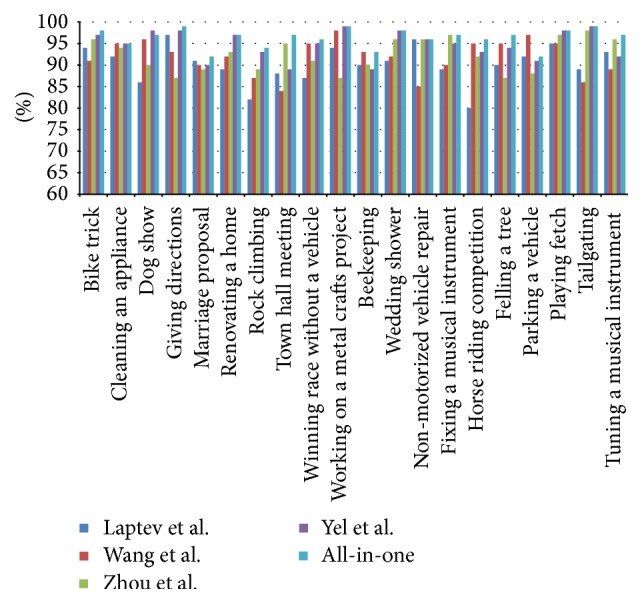
Compare proposed method with other BoW based methods on TRECVID MED16 dataset.

As Figure 6 shows, our proposed method can better take advantage of the useful information among dense SIFT and Dense Trajectory features and get higher accuracy than the other methods for all events except “parking a vehicle” and “dog show.” For “parking a vehicle” event, our method is more concerned with the actions of the human, but human action is very little in the car. For “dog show” event, Ye et al. use audiovisual bimodal and the barking of the dog gave more clues.

## 4. Conclusions

In this paper, we present using bimodel for extracting the relational information of local static feature and local motion feature. To overcome the weakness of BoW model, we further introduce a divisive algorithm to keep more information among feature discrete. Our experiments have shown that original static and motion features are complementary to their relational information.
